# Supplementary material for: Modelling opinion dynamics in the age of algorithmic personalisation
Source: Sci Rep. 2019 May 13;9:7261. doi: 10.1038/s41598-019-43830-2 (PMC6514165; doi:10.1038/s41598-019-43830-2)
Supplement: Supplementary file 1 — Supplementary Information [file 41598_2019_43830_MOESM1_ESM.pdf]

Modelling opinion dynamics in the age of algorithmic personalisation

Nicola Perra[1] and Luis E C Rocha[1]
[1]*Centre for Business Network Analysis, Business School,*
University of Greenwich, SE10 9LS, London, United Kingdom

Here, we provide supplemental information about the algorithmic filtering methods, the opinion dynamics model, and we present a sensitivity analysis to study the robustness of the results to the variation of the main parameters.

A. Algorithmic Personalization Mechanisms

Due the limited attention of each node, we assume that the social platform, where interactions take place, shows to each user a maximum of Q posts. Consider a node i. It is characterized by an activity p_i which determine the rate at which it engages with the social platform. This is extracted from a distribution $F(p)$. In the main, text we considered an heterogenous distribution, in section C we consider the case of a constant activity across the whole network. Suppose that at time t node i holds opinion A which it was adopted by looking at the posts contained in its timeline, $R_i(t_{last})$, the last time (t_{last}) it was active. Suppose that the node is now active. It first broadcasts its opinion to all its neighbors (which might or might not see it due the filtering mechanism). Between time t_{last} and t some of its neighbors might have been active and broadcasts their opinions. These go to an hidden list $L_i(t)$ which contains also the posts in $R_i(t_{last})$. In case the list $L_i(t)$ is larger than Q, some posts are not shown to the user and deleted. In fact the user will just see posts in its timeline $R_i(t)$. The sorting process is defined by the algorithm personalization mechanism under consideration. We considered four possible approaches:

- posts are selected randomly. This is the reference method (REF for short)
- posts are ordered considering the time when they were posted and the latest Q are selected. We call this method REC, that stands for recent
- posts are ordered considering the time when they were posted and the oldest Q are selected. We call this method OLD, as older posts are selected first
- posts are ordered considering the current opinion of the user which was formed by looking at the posts in its timeline and was the subject of a post at time t. This mimic semantic filtering methods as the filtering preferentially show to the user similar posts respect to what it recently broadcasted. We call this method PR for short.

Each method selects Q posts from the list $L_i(t)$, thus in general $|R_i(t)| \leq Q$.

B. Opinion Dynamics Model

After the algorithmic filtering, the node i is presented with a curated timeline $R_i(t)$. Remember that we considered a bipartisan system in which nodes can adopt one of two opinions, i.e. A or B. Thus the timeline, generally, contains $a|R_i(t)|$ posts in favor of A and $b|R_i(t)|$ in favor of B. The user will then adopt opinion A with probability a and opinion B with probability b. Thus, as mentioned in the main text the adoption process, although biased towards the majority, is not deterministic. As long a and/or b are greater than zero the user can adopt the two opinions.

C. Sensitivity Analysis

Below, we will show the results for i) constant activation rates ii) longer observation times iii) different Q sizes iv) more unbalanced initial conditions v) computing the $\langle P_A^{nn} \rangle$ distributions in the neighbourhood of each node i conditioning on the the opinion of i.

1. *Constant activation rates*

In the main text, we considered heterogenous activity patterns following a distribution $F(p) \sim p^{-1.5}$ with $p \in [0.01, 1]$. In these settings, the average activity $\langle p \rangle = 0.1$. In this section, we show the results considering that each node has the same activity $p = 0.1$. In Figure 1, we show the behavior of P_A as function of time for all the network topologies. In the first row, we show the results for a balanced initial condition in which $P_A(0) = P_B(0) = 0.5$, in the second row instead $P_A(0) = 0.2$ and $P_B(0) = 0.8$. The results are in qualitatively in line with what shown in the main text: none of the sorting mechanisms is able to break the status quo in case of balance initial conditions. Instead in the case of unbalanced initial start the PR mechanism reduces the fraction of the subordinate opinion. Furthermore, the largest reduction happens in the case WS networks with $g = 1$. Conversely, high values of the clustering coefficient, independently of the average shortest path, hamper the reduction as well as heterogenous connectivity patterns.

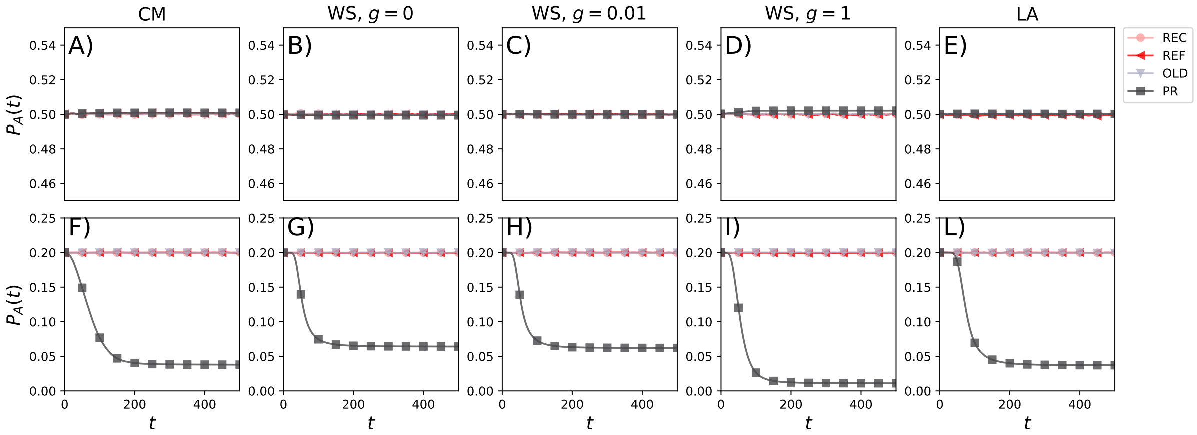

FIG. 1: Evolution of group opinion in the various network models. The prevalence $P_{\mathrm{A}}(t)$ of opinion A over time for starting from $P_{\mathrm{A}}(0) = 0.5$ and $P_{\mathrm{B}}(0) = 0.5$ (first row) and $P_{\mathrm{A}}(0) = 0.2$ and $P_{\mathrm{B}}(0) = 0.8$ (second row). Each column describes the results for one of the networks. In each plot, we show results for the four sorting algorithms. Each plot is the average of 10^2 independent simulations and to improve the visualization we are showing the data points every 50 time steps. In all scenarios we set $Q = 20$ and assume that each node as a constant activation probability $p = 0.1$.

In Figure 2, we show the distribution of the fraction of neighbours holding opinion A $\langle P_A^{nn} \rangle$. In order to highlight the difference respect to the initial conditions, on the y-axis we divided the distribution values at $t = 500$ for the same quantity computed at $t = 0$, which is then normalized by the initial conditions. The results are in line with what shown in the main text for heterogenous activity patterns. In particular, for a balanced starting condition (first row in the figure) both topological (WS with $g = 0$ and $g = 0.01$) induce the emergence of polarization effects. These are particularly visible for the semantic filtering (PR). Absence of clustering and heterogeneity in connectivity patterns are not conductive for such phenomena to emerge. For unbalanced initial conditions (second row in the Figure), the effects of clustering are much stronger. Semantic filtering induces the formations of echo chambers around the dominant opinion across all network topologies. However, as shown in the main text, heterogeneity in the connectivity patterns hamper this tendency.

2. *Longer running times*

In the main text, we run the simulations considering $T_{max} = 500$. We choose this value because the fraction of nodes holding opinion A (or B) seemed to reach an equilibrium. Here we test this assumption by considering $T_{max} = 5000$. In particular, we investigate the same scenarios plotted in Figure 2 in the main text and run for 10 times longer. As clear from Figure 3 the system reaches indeed an equilibrium that is stable (at least until 5000 time steps).

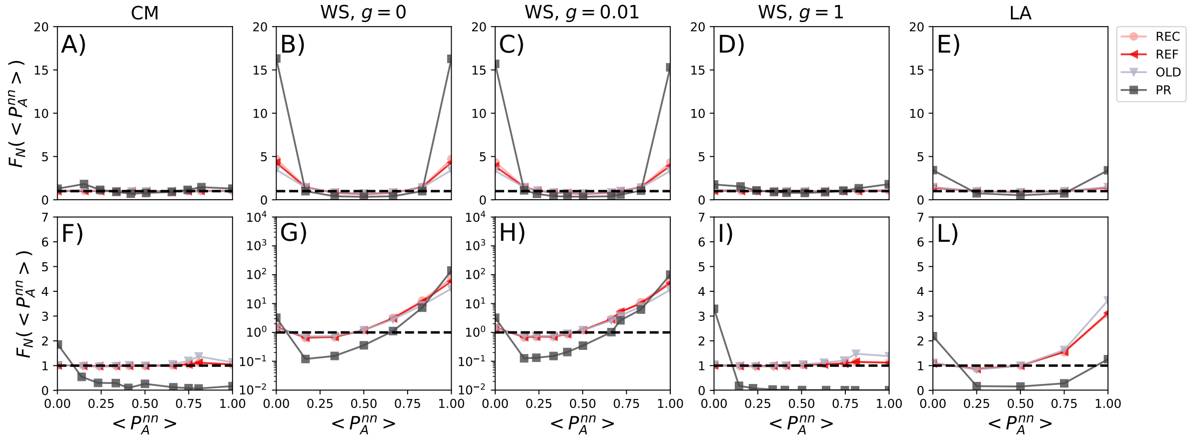

FIG. 2: Opinions of friends. We show the distribution of the fraction of friends (nearest neighbors, nn) of i with the same opinion A at $t = 500$. We normalize the y-axis by dividing for the same quantity computed at $t = 0$. In the first row we show the results for starting conditions $P_{\rm A}(0) = 0.5$ and $P_{\rm B}(0) = 0.5$. In the second row, for starting conditions $P_{\rm A}(0) = 0.2$ and $P_{\rm B}(0) = 0.8$. Each column described the results for a particular network. Each plot is the average of 10^2 independent simulations. In each plot, we consider the four ranking algorithms and set $Q = 20$ and assume that each node as a constant activation probability $p = 0.1$.

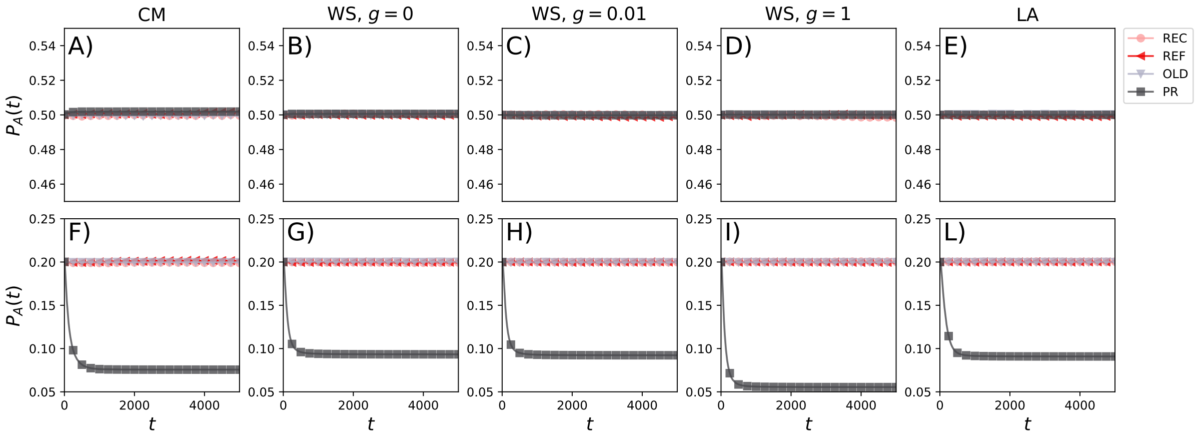

FIG. 3: Evolution of group opinion in the various network models. The prevalence $P_{\rm A}(t)$ of opinion A over time for starting $P_{\rm A}(0) = 0.5$ and $P_{\rm B}(0) = 0.5$ (first row) and $P_{\rm A}(0) = 0.2$ and $P_{\rm B}(0) = 0.8$ (second row). Each column describes the results for one of the networks. In each plot, we show results for the four sorting algorithms. Each plot is the average of 10^2 independent simulations. In all scenarios we set $Q = 20$.

3. Different queue sizes

We now turn to study the role of the queue sizes Q. In Figure 4 we show, for initial balanced conditions, the normalized distribution of the fraction of neighbors holding opinion A for $Q = 20$ (first row), $Q = 5$ (second row), and $Q = 50$ (third row). The results are qualitatively similar for all the values of Q. There are some differences in the case of small queue sizes ($Q = 5$) in case of high clustered topologies (WS networks with $g = 0$ and $g = 0.01$). Here, despite all the sorting mechanisms give rise to polarization, their efficiency is a bit different than for larger values of Q. Indeed, for $Q = 5$ the methods REF and REC, that provide either a random sample or a the latest posts, produce larger values of polarization than the PR method. Intuitively this is due to the fact that in case Q is small than the average degree of nodes (which in for WS networks is 6) the PR mechanism might reduce the polarization effects. To understand this effect think about the extreme situation in which nodes have all $Q = 1$. Consider a node i connected with 5 nodes having opinion A and only one with opinion B. Assume that i holds opinion B. In case $Q = 1$ has long

as there is at least one neighbor with the same opinion the node will not change to A. Instead for larger values of Q, despite the bias, the opinion A is likely to be more represented and eventually lead to a formation of cluster around that opinion.

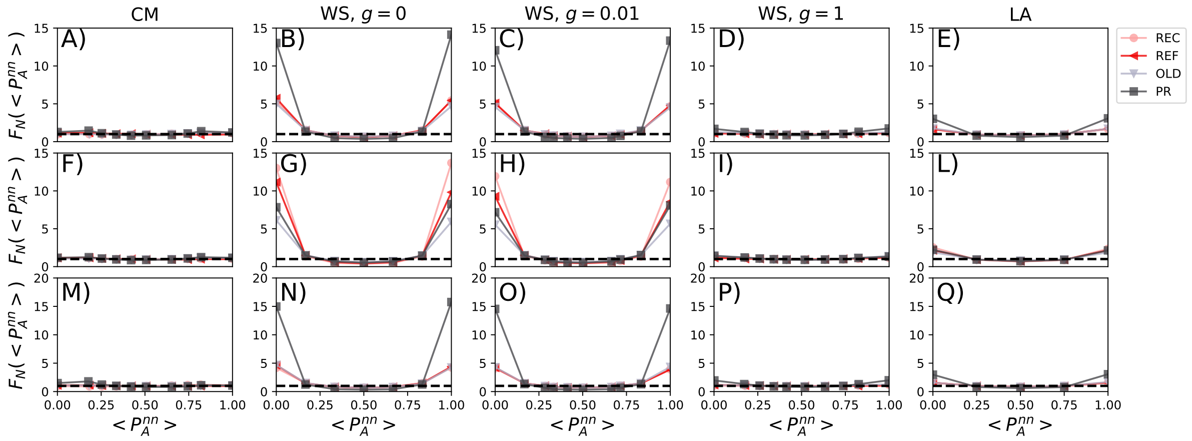

FIG. 4: Opinions of friends. We show the distribution of the fraction of friends (nearest neighbors, nn) of i with the same opinion A at $t = 500$. We normalize the y-axis by dividing for the same quantity computed at $t = 0$. We show the results for starting conditions $P_\mathrm{A}(0) = 0.5$ and $P_\mathrm{B}(0) = 0.5$. In the first row, we show the results for $Q = 20$, in the second for $Q = 5$, in the third for $Q = 50$. Each column described the results for a particular network. Each plot is the average of 10^2 independent simulations.

In Figure 5 we show the same plot considering however a $20 - 80$ initial conditions. The results are in line with what discussed above and in the main text. In particular, topological and spatial correlation induce the formation of echo chambers around the subordinate opinion. Also in this case these effects are stronger for REC and REF filtering mechanism.

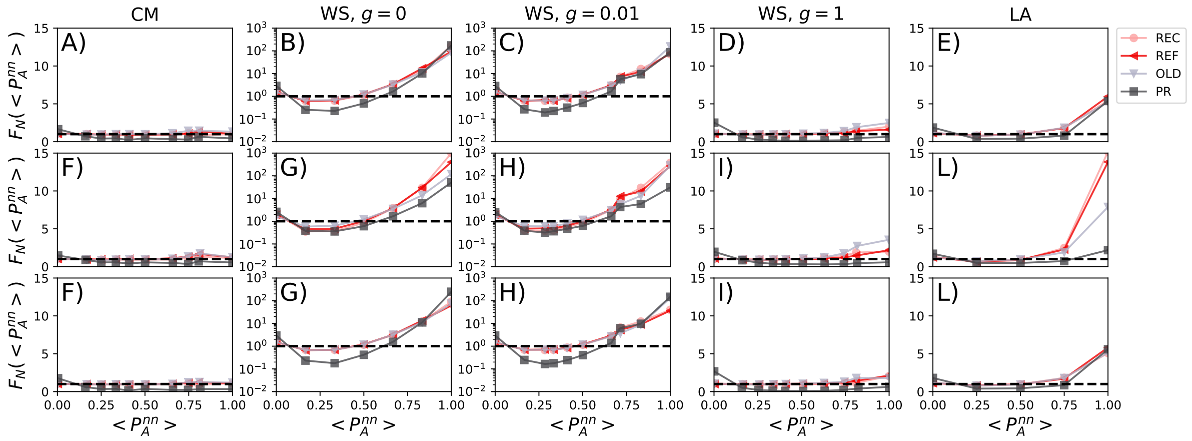

FIG. 5: Opinions of friends. We show the distribution of the fraction of friends (nearest neighbors, nn) of i with the same opinion A at $t = 500$. We normalize the y-axis by dividing for the same quantity computed at $t = 0$. We show the results for starting conditions $P_\mathrm{A}(0) = 0.2$ and $P_\mathrm{B}(0) = 0.8$. In the first row, we show the results for $Q = 20$, in the second for $Q = 5$, in the third for $Q = 50$. Each column described the results for a particular network. Each plot is the average of 10^2 independent simulations.

In Figure 6 we show the behavior of P_A as function of time for $Q = 5$. As clear from the figure, the behavior of the global share of the opinion is qualitatively similar to the other values of Q. For unbalanced initial conditions the semantic filtering is still the only sorting mechanism that bring to a reduction of the subordinate opinion. High clustering, spatial correlations, and heterogeneity in the degree distribution hamper the reduction respect to WS graphs with $g = 1$ however to a less extent than for $Q = 20$.

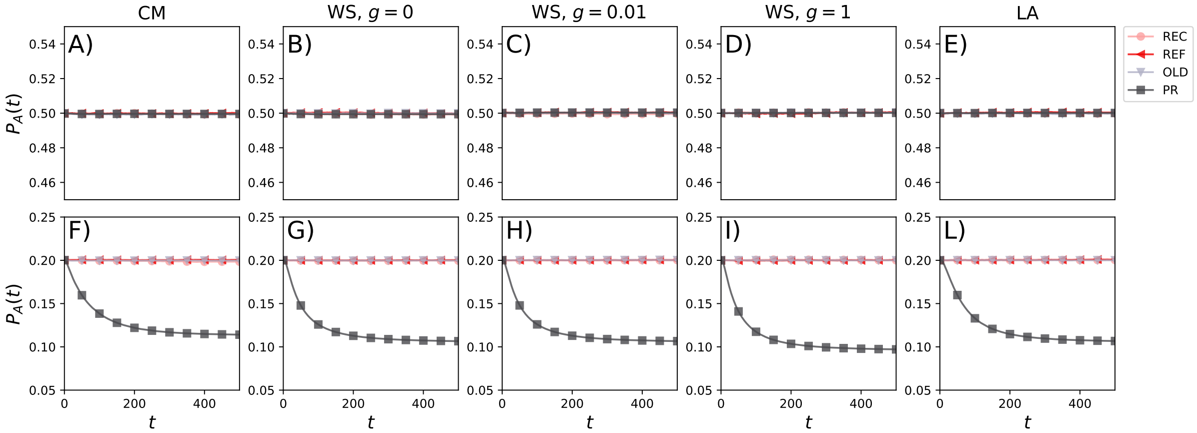

FIG. 6: Evolution of group opinion in the various network models. The prevalence $P_{\mathrm{A}}(t)$ of opinion A over time for starting from $P_{\mathrm{A}}(0) = 0.5$ and $P_{\mathrm{B}}(0) = 0.5$ (first row) and $P_{\mathrm{A}}(0) = 0.2$ and $P_{\mathrm{B}}(0) = 0.8$ (second row). Each column describes the results for one of the networks. In each plot, we show results for the four sorting algorithms. Each plot is the average of 10^2 independent simulations and to improve the visualization we are showing the data points every 50 time steps. In all scenarios we set $Q = 5$.

4. $1 - 99$ and $10 - 90$ initial conditions

We now consider the behavior of $P_A(t)$ for different unbalanced conditions. In Figure 7 we show (first row) the case of a quite unbalanced starting scenario: $P_A(0) = 0.01$ and $P_B(0) = 0.99$. As the opinion model we consider is not based on a deterministic majority rule, the subordinate opinion is able to survive. Also in this case, the PR mechanisms induces a reduction which is however hampered by correlations and heterogenous connectivity patterns as shown in the main text for $20 - 80$ case. In the second row we show the case in which $P_A(0) = 0.1$ and $P_B(0) = 0.90$ which provides the same qualitatively results.

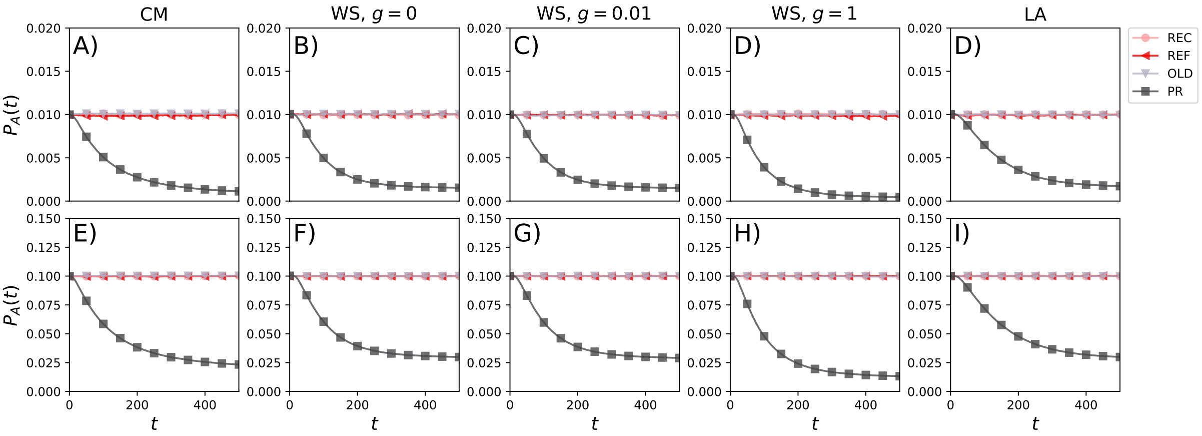

FIG. 7: Evolution of group opinion in the various network models. The prevalence $P_{\mathrm{A}}(t)$ of opinion A over time for starting from $P_{\mathrm{A}}(0) = 0.01$ and $P_{\mathrm{B}}(0) = 0.99$ (first row) and $P_{\mathrm{A}}(0) = 0.1$ and $P_{\mathrm{B}}(0) = 0.9$ (second row). Each column describes the results for one of the networks. In each plot, we show results for the four sorting algorithms. Each plot is the average of 10^2 independent simulations and to improve the visualization we are showing the data points every 50 time steps. In all scenarios we set $Q = 20$.

5. *Conditional* $\langle P_A^{nn} \rangle$ *and* $\langle P_B^{nn} \rangle$

In the main text (and above), we showed the distributions of opinions in the neighbourhoods of each node i. The computation was done irrespective of the opinion of i. In doing so, we were able to show in a single plot, possible polarisation around both opinions A and B. In this section instead, we show the conditional distributions: the distribution of neighbours holding opinion A (or B) for each node i holding opinion A (or B). In Figure 8 we show the correspondent of Figure 3 in the manuscript: $Q = 20$ and two initial distribution of the two opinions. As clear from the figure the plot is very similar to what we observed without conditioning on the opinion of each focal node. In particular, the presence of strong clustering in WS ($g = 0$ and $g = 10^{-2}$) induces the strongest polarisation. To a lesser extent this is also the case for spatial clustering (LA). The only difference, by conditioning on the opinion hold by each focal node, is visible in the case of unbalanced initial conditions for CM and WS ($g = 1$) networks. In fact, we see that respect to $T = 0$ there is increase (although much smaller than the other networks) of the polarisation. Interestingly, the PR mechanism is the one inducing the largest effects. In case the distributions are calculated without conditioning on the opinion of the focal node, this phenomenon is not observed as there is a general reduction of nodes holding opinion A at $T = 500$ respect to $T = 0$. Considering instead the subset of neighbours around the minority of nodes holding opinion A show the re-organisation of the network in compact domains that allow the minority to survive. In Figure 9 we show the correspondent of Figure 6 in the manuscript: $Q = 20$, two initial conditions in presence

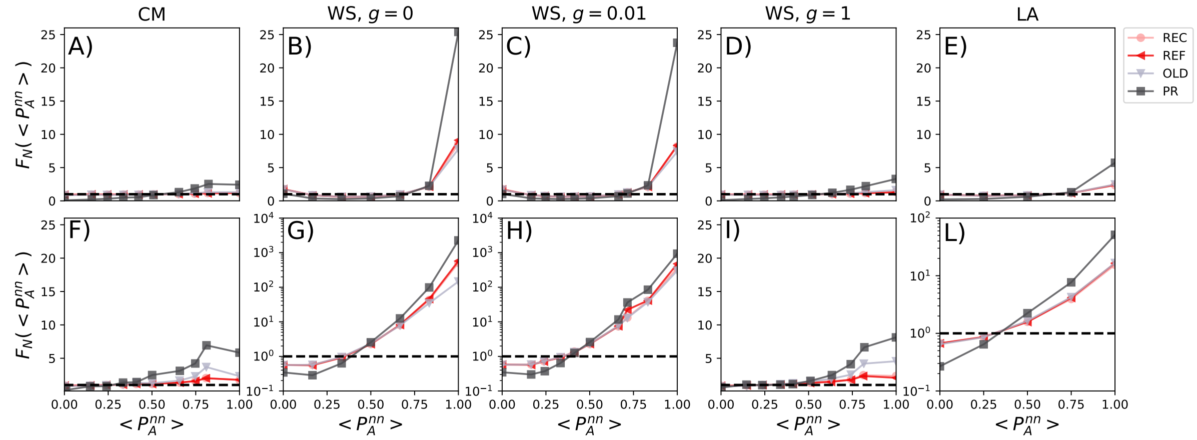

FIG. 8: Opinions of friends. We show the distribution of the fraction of friends (nearest neighbors, nn) of i holding opinion A at $t = 500$. We consider only the nodes i holding opinion A. We normalize the y-axis by dividing for the same quantity computed at $t = 0$. In the first row we show the results for starting conditions $P_{\rm A}(0) = 0.5$ and $P_{\rm B}(0) = 0.5$. In the second row, for starting conditions $P_{\rm A}(0) = 0.2$ and $P_{\rm B}(0) = 0.8$. Each column described the results for a particular network. Each plot is the average of 10^2 independent simulations. In each plot, we consider the four ranking algorithms and set $Q = 20$.

of nudging. The behaviour observed is very similar even conditioning on the opinion of each focal node. Finally, in Figure 10 we show $\langle P_B^{nn} \rangle$ the distribution of neighbours holding opinion B in case the focal node has opinion B, for $Q = 20$, two initial conditions in presence of nudging ($z = 0.01$). The figure clearly confirms the picture emerging from the main text and the previous plot.

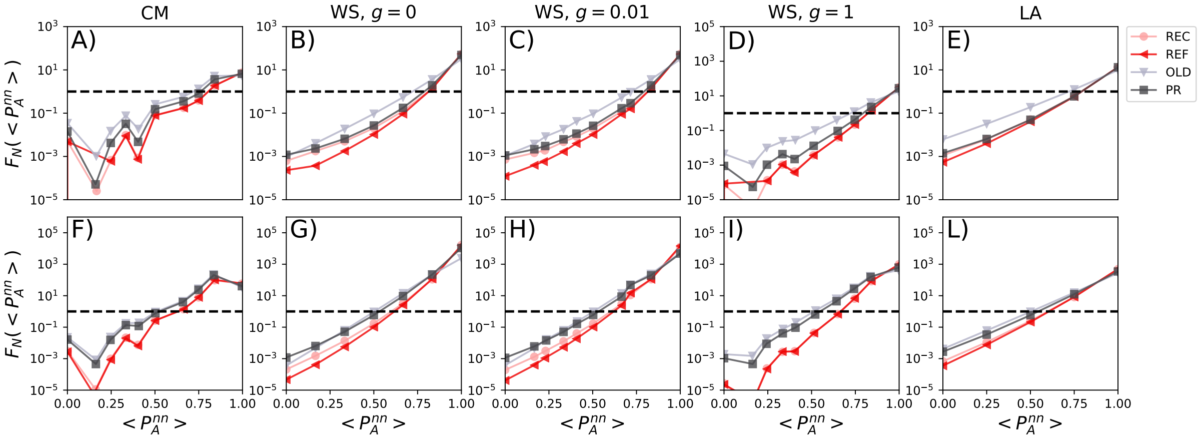

FIG. 9: Opinions of friends in a nudging scenario with $z = 0.01$. We show the distribution of the fraction of friends (nearest neighbors, nn) of i holding opinion A at $t = 500$. We consider only the nodes i holding opinion A. We normalize the y-axis by dividing for the same quantity computed at $t = 0$. In the first row we show the results for starting conditions $P_{\rm A}(0) = 0.5$ and $P_{\rm B}(0) = 0.5$. In the second row, for starting conditions $P_{\rm A}(0) = 0.2$ and $P_{\rm B}(0) = 0.8$. Each column described the results for a particular network. Each plot is the average of 10^2 independent simulations. In each plot, we consider the four ranking algorithms and set $Q = 20$.

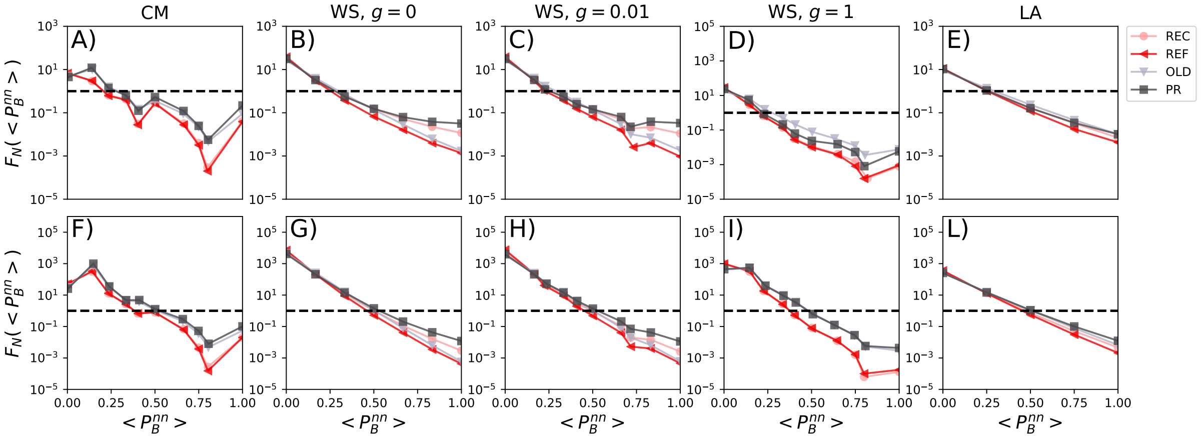

FIG. 10: Opinions of friends in a nudging scenario with $z = 0.01$. We show the distribution of the fraction of friends (nearest neighbors, nn) of i holding opinion B at $t = 500$. We consider only the nodes i holding opinion B. We normalize the y-axis by dividing for the same quantity computed at $t = 0$. In the first row we show the results for starting conditions $P_{\rm A}(0) = 0.5$ and $P_{\rm B}(0) = 0.5$. In the second row, for starting conditions $P_{\rm A}(0) = 0.2$ and $P_{\rm B}(0) = 0.8$. Each column described the results for a particular network. Each plot is the average of 10^2 independent simulations. In each plot, we consider the four ranking algorithms and set $Q = 20$.
